# Differentiation of Human Mesenchymal Stem Cells into Corneal Epithelial Cells: Current Progress

**DOI:** 10.3390/cimb46120792

**Published:** 2024-11-21

**Authors:** Abdul Malik Setiawan, Taty Anna Kamarudin

**Affiliations:** 1 Department of Anatomy, Faculty of Medicine, Universiti Kebangsaan Malaysia, Cheras, Kuala Lumpur 56000, Malaysia; abdul.malik.setiawan@kedokteran.uin-malang.ac.id; 2 Department of Anatomy, Maulana Malik Ibrahim State Islamic University, Malang 65144, Indonesia

**Keywords:** mesenchymal stem cell, differentiation, co-culture, BMP4, retinoic acid, hTCEpi

## Abstract

The limited availability of corneal tissue grafts poses significant challenges in the treatment of corneal blindness. Novel treatment utilizes stem cell grafts transplanted from the healthy side of the cornea to the damaged side. However, this procedure is only possible for those who have one-sided corneal blindness. Human stem cells offer promising potential for corneal tissue engineering, providing an alternative solution. Among the different types of stem cells, mesenchymal stem cells (MSCs) stand out due to their abundance and ease of isolation. Human MSCs can be derived from bone marrow, adipose, and umbilical cord tissues. Differentiating MSC toward corneal tissue can be achieved through several methods including chemical induction and co-culture with adult corneal cells such as human limbal epithelial stem cells (LESCs) and human corneal epithelial cells (hTCEpi). Adipose-derived stem cells (ADSCs) are the most common type of MSC that has been studied for corneal differentiation. Corneal epithelial cells are the most common corneal cell type targeted by researchers for corneal differentiation. Chemical induction with small molecules, especially bone morphogenetic protein 4 (BMP4), all-trans retinoic acid (ATRA), and epidermal growth factor (EGF), has gained more popularity in corneal epithelial cell differentiation. This review highlights the current progress in utilizing MSCs for corneal differentiation studies, showcasing their potential to revolutionize treatments for corneal blindness.

## 1. Introduction

Corneal blindness is a major public health concern worldwide, affecting millions of people [1]. Etiological causes of corneal blindness can be categorized into infection, dystrophies, and degeneration of corneal tissue. Most of the causes are preventable, but undertreatment can lead to irreversible damage and cause visual impairment. Corneal blindness can be prevented through early intervention of the etiological cause. However, failed treatment or therapy for certain conditions being unavailable might lead to corneal opacity and scarring [2].

The most common treatment for corneal blindness is corneal transplantation using donated human corneal tissue [3]. However, the shortage of corneal donors and the risk of graft rejection have limited the success of this approach [4]. Corneal tissue differentiation using stem cells has been proposed as a potential alternative to human donors as they are abundant and easily accessible [5].

Stem cells are a type of cell that has the ability to differentiate into specialized cells or tissue and renew themselves in order to maintain the homeostasis of the tissue. Because of their potential in regenerative medicine, research on stem cells has become increasingly popular and has been developing over the last 50 years [6]. This review is dedicated to analyzing the current advancements in stem cell differentiation research, specifically focusing on the methods used to transform human mesenchymal stem cells into corneal epithelial cells and their outcomes as an alternative therapy for corneal blindness.

## 2. Corneal Blindness and Current Treatment

**Figure 1:**
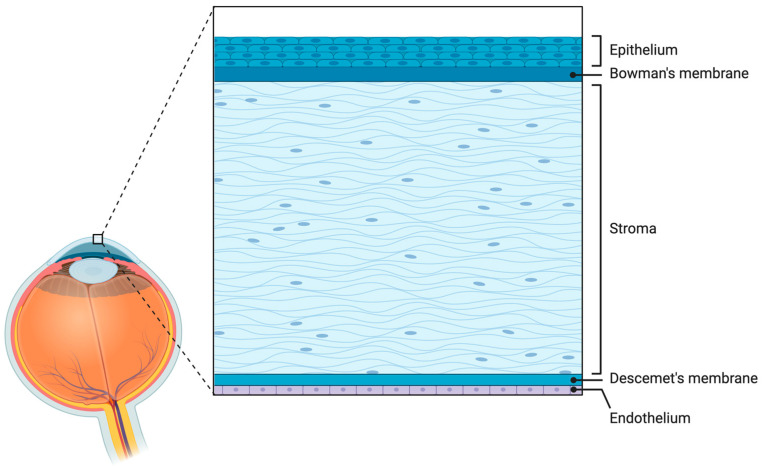
The five layers of the cornea. This figure illustrates the cross-sectional view of corneal tissue. The layers are as follows: the epithelium, Bowman’s membrane, stroma, Descemet’s membrane, and endothelium. This schematic was created with BioRender.com.

According to the World Health Organization (WHO), corneal blindness is a visual impairment with visual sharpness of 3/60 or less [7]. The corneal layer consists of endothelial cells, Descemet’s membrane, stroma, Bowman’s layer, and the epithelium. As presented in Figure 1, the corneal epithelial layer is located in the outermost layer while the corneal endothelial layer is located in the innermost layer of the cornea [8]. Any pathological mechanism that disrupts the transparent layer of the epithelium, stroma, and endothelium layer could lead to corneal blindness [2]. The anterior part of the cornea commonly becomes damaged by traumatic insults such as lacerations or chemical injury; often leading to visual impairment [9]. In 2015, corneal blindness caused by corneal opacity and trachoma accounted for 4% of the global causes of visual loss among adults aged 50 years and older [1].

### 2.1. Epidemiology of Corneal Blindness

Between 1990 and 2015, corneal disease accounted for at least 2.4% of blindness and visual impairment in South and East Asia [2,7]. By 2020, it was estimated that 0.4% of people across Asia with moderate-to-severe visual impairment (MSVI) and blindness were affected by corneal disease [10]. Additionally, it was estimated that 5.5 million people worldwide were bilaterally blind due to corneal opacity in 2020 [11]. There are multiple diseases that lead to corneal blindness, and the disease varies between countries. Fuchs dystrophy in the USA, keratoconus in Australia, and trachoma in Africa are types of corneal diseases that make patients seek surgical interventions [12].

Infectious keratitis due to bacteria, viruses, fungi, and protozoa, frequently worsened by factors such as trauma or contact lens misuse, could progressively develop into permanent corneal scarring and blindness [13]. Bacterial keratitis is the most prevalent form of infectious keratitis, with *Staphylococci* species being the primary microorganisms responsible [14]. Additionally, recurrent conjunctival infections caused by Chlamydia trachomatis, known as trachoma, lead to conjunctival scarring and cause the eyelashes to rub against the corneal surface. The combination of chronic inflammation and repeated corneal trauma can result in permanent corneal scarring and blindness [15]. Trachoma is widespread in many of the poorest and most rural areas of Africa, Central and South America, and Asia [16].

### 2.2. Current Treatment for Corneal Blindness

Early stages of medical treatment for corneal surface injury could prevent corneal blindness [17]. However, when scarring and opacity in the cornea have already occurred, the most effective treatment is corneal transplantation or keratoplasty. In addition, corneal transplantation is considered the most common type of transplantation in the world. Although corneal transplantation has been adopted around the world, the availability of corneal grafts from deceased donors constrains efforts to provide treatment for the disease. In 2012, less than 2% of patients who needed corneal transplantation actually underwent the surgery. From around 116 countries that perform corneal transplantation, only 82 countries actually supply the tissue [3]. It shows that corneal graft is currently in high demand due to its limited supply.

## 3. Stem Cells in the Current Treatment of Corneal Blindness

Acute or chronic injury that destroys the limbal area of the eye could lead to unilateral or total limbal stem cell deficiency (LSCD). In the case of corneal blindness that is caused by LSCD, the gold standard treatments are autologous transplantation of limbal stem cells (LSC) harvested from the patient’s own healthy eye or allotransplantation LSC harvested from a healthy donor [18]. A systematic review of 13 years of limbal stem cell transplantation by Baylis and colleagues proves that limbal cell transplantation has a high success rate in patients with limbal cell deficiency [19]. However, the availability of donated corneal grafts is mostly limited. Moreover, the use of autologous LSC transplantation is only available for patients with unilateral LSCD, since patients with bilateral LSCD can only receive allotransplantation.

In order to overcome this limitation, autologous mesenchymal stem cells can be used as an alternative to allogeneic corneal stem cell sources. Holan et al. in 2015 showed that bone marrow and adipose tissue mesenchymal stem cells (MSCs) had similar therapeutic effects to LSC in the treatment of corneal surface injuries in animals [20]. Thus, it was suggested that MSC-derived corneal grafts be used as a therapeutic source for patients with bilateral LSCD. On the other hand, a systematic review by Sanabria-de la Torre et al. in 2021 also reported that mesenchymal stem cells (MSCs) have regenerative properties to produce ex vivo differentiated cells or tissues and proved to be safe because of their hypoimmunogenicity, even for allogenic therapies [21].

### 3.1. A Brief History of Stem Cells

Stem cell research started with pluripotent stem cell (PSC) isolation from mouse bone marrow cells in 1961, followed by human embryonic stem cell (hESC) isolation in 1998, human adipose-derived stem cells (hADSCs) in 2001, and induced pluripotent stem cells (iPSCs) by reprogramming adult somatic cells in 2006 [22,23,24]. However, currently, only hematopoietic-based stem cell therapy has received government approval for clinical application. This is due to the potential risk of teratogenesis and immunological graft rejection that is still not fully understood [25,26].

Yamanaka was the first to reprogram adult human cells into iPSCs in 2007. Following this breakthrough, Nishida and his team were able to generate corneal epithelial cells from human iPSCs and successfully transplanted them into a rabbit in 2016. The first human clinical application was performed in 2019 by Nishida and his team. They successfully transplanted the synthetic corneal epithelial cells to the ocular surface of a patient with limbal stem cell deficiency [24,27]. The limited clinical application of stem cell treatment has encouraged many researchers to expand the potential usage of stem cells such as in the development of synthetic biomaterial for tissue engineering.

### 3.2. Different Types of Stem Cells

Based on their differentiation potential, stem cells are classified as totipotent, pluripotent, multipotent, oligopotent, and, unipotent. Totipotent stem cells found in a zygote have the highest potency and can differentiate into embryonic as well as extra-embryonic cells [28]. Pluripotent stem cells found in hESCs can differentiate into any type of somatic cell in the body except the placenta [29,30]. Multipotent, oligopotent, and unipotent are types of stem cells that can only differentiate into somatic stem cells within specific lineages, specific tissue, or even specific types of cells [31].

Based on the time of discovery, hESCs were first isolated in 1998, followed by human-induced pluripotent stem cells and multipotent and unipotent adult stem cells [30]. Unipotent adult stem cells are undifferentiated stem cells that populate specific tissues or organs such as the skin, muscle, neural tissue, and blood. The function of these so-called stromal cells is to renew the damaged cells in the specific tissue or organ [6].

Different approaches in stem cell treatment draw certain limitations that constrain mass adoption in clinical practice. Ethical consideration is one of the main factors that limits the adoption of hESC treatment. Takahashi and team propose that reverse engineering of adult somatic cells into pluripotent stem cells is a promising alternative to hESC [23]. However, there are many debates on whether iPSCs have similar pluripotency compared to hESCs. In addition, a recent study suggests that iPSCs may pose a risk of teratoma formation [32].

Another potential source of stem cell treatment that is gaining popularity is mesenchymal/stromal stem cells (MSCs). MSCs have become the most popular treatment in regenerative cell therapy because of their efficient cost and abundance in supply. However, MSC procurement from certain locations such as the bone marrow compartment is difficult. Since then, other sources of autologous multipotent stem cells have started to emerge, namely hematopoietic, neural, endothelial, and adipose sources of stem cells [6].

## 4. Mesenchymal Stem Cells (MSCs) in Corneal Differentiation Studies

An MSC is a type of multipotent stromal cell that can differentiate into a variety of cell types, including osteoblasts, chondrocytes, myocytes, and adipocytes [33]. These cells are characterized by their spindle-shaped morphology and the ability to produce an extracellular matrix rich in hyaluronic acid. MSCs play a crucial role in tissue repair and regeneration, and they have significant potential in regenerative medicine due to their immunomodulatory effects and their capacity to differentiate into various cell types [34]. Initially discovered in bone marrow, MSCs have since been isolated from a variety of tissues such as adipose tissue, umbilical cord blood, and dental pulp [33].

### 4.1. Identification of MSCs

Upon procurement from human tissue, isolated human MSCs can be evaluated using visual identification with microscopy, cell shorting analysis with flow cytometry, gene expression with real-time PCR, and biochemical assays with an antigen–antibody protocol. Confocal, bright-field, and fluorescent microscopy are the most common techniques to visually identify and quantify human MSCs. Human MSCs present different morphologies according to the source of tissue. For example, MSCs isolated from subcutaneous adipose tissue have a fibroblast-like shape with a small diameter, while MSCs isolated from visceral adipose tissue have a rounded form similar to epithelial cells [35].

In 2016, the International Society for Cellular Therapy released guidelines for minimal criteria for human MSC identification. MSCs must be plastic adherent, express cell surface markers of CD44, CD73, CD90, and CD105 negative for hematopoietic markers including CD45 and CD34, and be able to be differentiated into adipocytes, chondrocytes, and osteocytes [33]. In addition to this, HLA-ABC positive expression alongside HLA-DR negative expression can also be used in flow cytometry assays [36]. Multipotent cells isolated from adipose tissue must adhere to these specific criteria to be categorized as mesenchymal stem cells (MSCs).

### 4.2. Different Types of MSCs

MSCs can be isolated from several human tissues such as the umbilical cord, bone marrow, and fat tissue. MSCs isolated from bone marrow and adipose tissue are the most frequently used sources in MSC research. These tissues, which are considered either renewable (like bone marrow) or surplus (like adipose), can be harvested from humans. Additionally, two “adult” tissues that are typically discarded after birth, the umbilical cord tissue and placenta, are also popular sources of human MSCs. Other sources of MSCs are skeletal muscle, dental pulp, fetal blood, and lung and heart tissues [34].

#### 4.2.1. Bone Marrow-Derived MSCs (BM-MSCs)

Bone marrow mesenchymal stem cells (BM-MSCs) are multipotent stem cells that can be isolated from bone marrow tissue. As part of mesenchymal stem cells, BM-MSCs should follow minimal criteria for MSCs such as plastic adherence and having the capacity of multilineage differentiation into osteocytes, adipocytes, and chondrocytes. BM-MSCs should also express specific surface markers such as CD73, CD90, and CD105 while lacking hematopoietic markers like CD34, CD45, and CD14. Genotypically, they maintain a stable karyotype through extensive culture and exhibit specific gene expression profiles related to mesenchymal lineage differentiation [33,34]. Generally, MSCs retain their specific gene expression until passages 4 to 6 and maintain this phenotypic profile for about 20 to 30 population doublings [37,38].

Recent studies have demonstrated the potential of BM-MSCs in tissue engineering applications, such as the repair of cartilage defects and bone regeneration, through their ability to modulate the immune response and promote tissue repair. BM-MSCs can be delivered using different types of scaffolds, such as decellularized extracellular matrix scaffolds, synthetic scaffolds, and nanomaterial-based scaffolds. They have the ability to adhere to the scaffold, proliferate, and differentiate into osteoblasts, promoting bone formation. The combination of BM-MSCs and scaffolds can enhance the efficacy of bone repair and regeneration [39]. BM-MSCs also possess regenerative and immunomodulatory properties that can enhance corneal wound healing, reduce inflammation, and promote tissue regeneration. In addition, conditioned media from BM-MSCs contain the IL-1 receptor antagonist (IL-1RA), which promotes wound closure in corneal epithelial wounds in animals [40].

#### 4.2.2. Umbilical Cord Derived MSCs (UC-MSCs)

Umbilical cord-derived mesenchymal stem cells (UC-MSCs) are categorized as perinatal/fetal MSCs since they can only be obtained from the extraembryonic tissue of a baby at birth. UC-MSCs can be isolated from umbilical cord tissue, Wharton’s jelly, and amniotic membrane. UC-MSCs are considered to have the highest proliferation and differentiation capacity despite having a limited time span. Different from adult MSCs like BM-MSCs and adipose-derived stem cells (ADSCs), UC-MSCs are free of aging factors as they come from the early age of the human form [41].

Wharton’s jelly MSCs (WJ-MSCs) are a type of UC-MSC obtained from the Wharton’s jelly portion of the umbilical cord. They have multilineage potential, immunomodulatory properties, and anti-fibrotic effects. WJ-MSCs also have low immunogenicity, making them suitable for allogeneic transplantation [42]. They have been used in clinical trials for conditions such as acute graft-versus-host disease, Parkinson’s disease, and acute myocardial infarction, with promising results [41].

#### 4.2.3. Adipose-Derived Stem Cells (ADSCs)

Adipose-derived stem cells (ADSCs) are one of the most abundant types of MSC and are easy to procure compared to other sources of MSCs such as BM-MSCs [43]. ADSCs are found in adipose tissues that are widely distributed throughout the body. In addition, ADSCs possess self-renewal and multipotency properties that make them a popular source of stem cells among researchers as the presence of adipose tissue in the human body is more abundant compared to bone marrow tissue. In Addition, adipose tissue contains more mesenchymal stem cells compared to bone marrow. Potentially, 1 g of adipose tissue contains approximately 5000–20,000 stem cells, whereas only about 10,000 can be potentially harvested from 1 g of bone marrow tissue [44]. ADSCs have drawn worldwide attention as a promising source for cell therapy in various pathologic conditions in corneal surfaces ranging from stem cell transplantation to corneal tissue engineering. Espandar et al. showed that human ADSCs prevent corneal damage in an animal model with alkaline burn injury [45].

Adipose tissue can be classified into two main types: subcutaneous adipose tissue (SAT) and visceral adipose tissue (VAT). SAT is located directly under the skin, while VAT is located around the organs in the abdominal cavity [46]. Studies have shown that VAT is linked with a higher risk of metabolic dysfunction, inflammation, and insulin resistance compared to SAT [47]. Additionally, it has been suggested that the location of adipose tissue may affect the characteristics of ADSCs, including their differentiation potential [48]. A study by Tang in 2017 showed that ADSCs isolated from VAT had greater proliferation capacity compared to ADSCs isolated from SAT. However, ADSCs isolated from SAT had greater differentiation capacity compared to ADSCs isolated from VAT [49].

Human ADSCs can be isolated from different locations in the human body. Most of the studies involving primary ADSCs involved ADSCs isolated from subcutaneous fat tissues. However, several studies have shown that ADSCs from different depots have different characteristics and functionality [50]. The differing expression of bone morphogenetic protein 4 *BMP4* between subcutaneous and visceral ADSCs suggests that adipose tissue depots may harbor distinct regulatory mechanisms governing their respective stem cell populations [51]. Depot-specific differences in the microenvironment, including variations in cell–cell interactions, paracrine signaling, and extracellular matrix composition, may contribute to the observed disparities in *BMP4* expression [50]. Understanding these depot-specific regulatory mechanisms could provide valuable insights into the functional variations in both subcutaneous and visceral adipose tissues and aid in the development of finely tailored regenerative therapies.

### 4.3. In Vitro Differentiation of MSCs into Corneal Epithelial Cells

**Figure 2:**
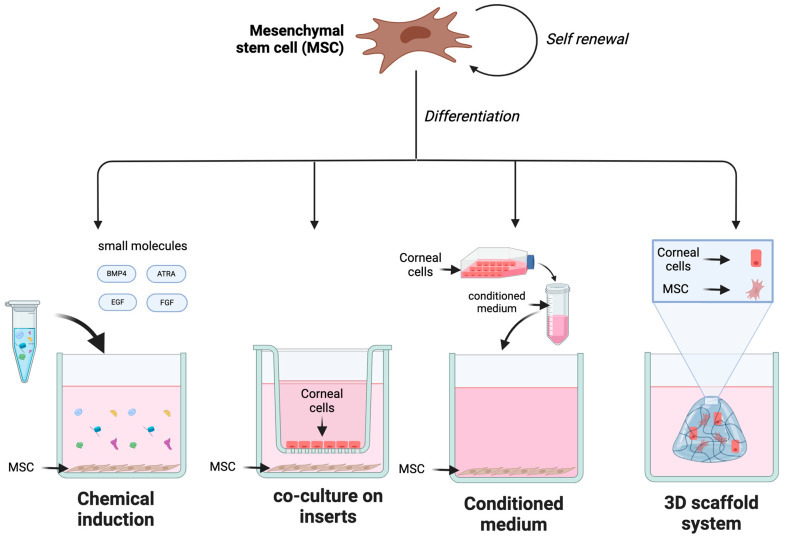
MSC differentiation method towardfor corneal differentiation. This figure illustrates the different methods of MSC differentiation into corneal cells. The methods include chemical induction with small molecules, co-culture on inserts with corneal cells, growth in a conditioned medium harvested from corneal cell culture, and cultivation of MSC and differentiated corneal cells within a 3D scaffold system. This schematic was created with BioRender.com.

Differentiation of human MSCs in vitro can be achieved through several methods as illustrated in Figure 2. Corneal differentiation can be induced by culturing the MSCs in a conditioned medium, a medium supplemented with signaling molecules only, or by co-culturing with the signal-providing cells via a cell culture insert or a 3D scaffold system [52,53]. In certain environments, MSCs isolated from mesodermal origin tissue can differentiate not only into mesodermal cell lineages such as osteoblast, chondrocytes, myocytes, and adipocytes but also into ectodermal and endodermal lineages. Trans-differentiation of MSCs is thought to be difficult, but studies have reported that under certain laboratory conditions, adipose-derived MSCs can undergo corneal differentiation and several other ectodermal lineages [54,55].

Differentiated human MSCs to cornea epithelial-like cells can be evaluated based on the expression of corneal epithelial cell surface markers. The expression of CK3, CK12, E-Cadherin, and PAX6 on the cell surface can be used to evaluate whether the differentiation of human ADSC has succeeded [56,57]. Other corneal cell markers, such as ZO-1, Na^+^ ATPase, AQP1, and N-cadherin, are used to identify differentiation toward corneal endothelial cells. Meanwhile, the expression of p63 and ABCG2 is more indicative of limbal epithelial stem cells [58,59,60].

#### 4.3.1. Chemical Induction Method

The chemical induction method utilizes small molecules and growth factors to induce the proliferation and differentiation of pluripotent cells. BMP4 is one of the small molecules that is used as a chemical inducer to differentiate MSCs into corneal epithelial cells, which are derived from the ectodermal lineage. Bone morphogenetic protein 4 (BMP4) is a member of the transforming growth factor beta (TGF-β) superfamily that regulates a variety of cellular processes, including differentiation, proliferation, and apoptosis [61]. BMP4 signaling is essential for the differentiation of ADSCs into corneal epithelial cells, and manipulating BMP4 signaling can enhance the efficiency of corneal epithelial tissue engineering [62,63].

BMP4 proteins activate both canonical and non-canonical TGF-β signaling pathways, leading to the differentiation of pluripotent cells toward the ocular surface of the ectodermal lineage [27]. TGF-β signaling pathways are activated when BMP4 dimers attach to a complex of type I (BMPRI) and type II (BMPRII) BMP receptors on the cell surface. This binding leads to the phosphorylation and activation of BMPRI by BMPRII, which then phosphorylates SMAD1, SMAD5, and SMAD8 to produce receptor-regulated SMADs (R-SMADs). R-SMADs then recruit SMAD4 to form Co-SMAD and translocate into the nucleus, leading to downstream signaling [64].

BMP4 has been used as a differentiation factor in several studies to differentiate ADSCs into an ectodermal origin cell, keratinocytes. Petry et al. and Maeda et al. used a combination of BMP4 (17 & 25 ng/mL) and 1.5 ng/mL all-trans retinoic acid (ATRA) to induce keratinocyte differentiation. Both studies successfully showed an increase in cytokeratin expression (CK1, CK8, CK10, and CK18) [54,65]. Bandeira et al. used a combination of 10 μM ATRA with valproic acid supplements to induce the differentiation of ADSCs into corneal epithelial cells. Both CK3 and CK12 gene and protein expressions were increased in the treatment group compared to the control group [66].

BMP4 and ATRA were also used to differentiate BM-MSC toward corneal epithelial cells. Katikireddy et al. used a combination of BMP4, RA, and epidermal growth factor (EGF), while Nieto-Nicolau et al. used only RA to differentiate BM-MSC toward corneal epithelial cells. Both studies showed an increase in CK3, CK8, and CK12 gene and protein expression [67,68]. Based on these findings, ectodermal differentiation of MSCs might not be activated only via the TGFβ signaling pathway but also through retinoic acid (RA) signaling pathways. This is in agreement with reports that suggest that the RA signaling pathway leads to Wnt signaling inhibition and promotes the non-neural ectodermal differentiation and proliferation of corneal epithelial cells [26,69,70]. Interestingly, Marta et al. in 2021 showed that inhibition of the TGFβ/Activin/Nodal and glycogen synthase kinase (GSK) 3 pathway was able to induce the differentiation of ADSCs toward corneal endothelial cells rather than corneal epithelial cells [71].

BMP4, epidermal growth factor (EGF), and retinoic acid are a few of the small molecules that are consistently used as supplements in MSC differentiation toward corneal epithelial cells. However, many studies have also successfully utilized various small molecules in induction methods other than BMP4 and ATRA in MSC differentiation toward corneal epithelial cells. Du et al. in 2010 were able to differentiate ADSCs into keratocytes, fibroblasts of the corneal stroma, using ascorbate and insulin as chemical inducers [72]. A few studies have supplemented epidermal growth factor (EGF) with other molecules to differentiate BM-MSCs and conjunctival MSCs toward corneal epithelial cells [73,74,75].

#### 4.3.2. Co-Culture Differentiation Method

The co-culture method in cell culture refers to a technique where multiple types of cells are cultured together in the same environment. Co-culture methods can be direct or indirect techniques. This method allows for cellular interactions, and it can impact the differentiation of ADSCs by receiving signals from neighboring cells. The in-direct co-culture method provides a physical barrier between cell types of interest. Semi-permeable barriers allow cell secretomes produced by the first cell to pass the membrane and interact with ADSCs to mimic paracrine activities in the human body [76].

The co-culture differentiation method for MSC differentiation toward corneal epithelial-like cells may not be as popular as the chemical induction method, but several studies have utilized the co-culture method to induce the differentiation of MSCs toward corneal epithelial cells as well as corneal stroma. Garzon et al., in 2014, successfully differentiated WJ-MSCs into corneal epithelial cells by co-culturing the MSCs with keratocytes in a hydrogel scaffold 3D system. Their method showed an increase in the protein expression of CK3, CK12, and CX43 [77]. In addition, Tsai et al. in 2015 showed that stem cells from human exfoliated decidua MSCs (HED-MSCs) can be differentiated into corneal epithelial cells by culturing them with a corneal epithelial cell line for 21 days while Sikora et al. in 2019 used the co-culture method to differentiate human ADSCs into LESCs by culturing the ADSC cell line with porcine LESCs. Both studies showed an increase in CK3 and CK19 gene and protein expression [78,79].

#### 4.3.3. Conditioned Media

Conditioned media are a type of cell culture media that have been conditioned with the cell culture environment of certain types of cells. Upon proliferation, cells release various bioactive molecules such as proteins, lipids, cytokines, mRNA, and growth factors to support cell replication [80]. Conditioned media can be prepared from differentiated adult cells or pluripotent stem cells. Large scalability, lower immune incompatibility, and lower tumorigenesis risk are several advantages of conditioned media-based therapy [81].

The conditioned media differentiation method utilizes bioactive molecules secreted from differentiated target cells. The culture media are then sterilized and added to the MSCs. An in vitro microenvironment containing conditioning signals secreted by differentiated human corneal epithelial cells can be used to induce the corneal differentiation of MSC. Nieto-Miguel et al., in 2013, were able to differentiate ADSCs into corneal epithelial cells by culturing ADSCs with conditioned media that carry secretomes from corneal epithelial cells [53].

#### 4.3.4. 3D Scaffold System

Stem cell differentiation using a 3D scaffold system involves biomaterial structures to provide an extracellular matrix (ECM) environment and facilitate the interactions between MSCs and the differentiated cell inducers. This application mimics the natural differentiation processes within the human tissue [82]. As mentioned before, a 3D scaffold system can be utilized to provide an extracellular matrix for the co-culture method [77]. Furthermore, a 3D scaffold system can also combined with chemical induction differentiation. Soleimanifar et al. in 2017 showed that the combination of nanofibrous scaffold and differentiation medium was effective in differentiating conjunctiva mesenchymal stem cells (CJ-MSCs) into corneal epithelial cells, which was shown by the increase in *CK3* gene expression compared to the control group [74]. In addition, Espandar et al. showed that a combination of hydrogel scaffolds and basic fibroblast growth factor (bFGF) could differentiate ADSCs toward corneal stroma-like cells [83].

### 4.4. Summary of the In Vitro Corneal Differentiation of MSCs

In vitro, the differentiation of human MSCs toward corneal tissue has been extensively studied in the last few decades. Most of the MSC sources used are AT-MSCs followed by BM-MSCs and UC-MSCs. MSCs isolated from human conjunctiva and dental pulp have also been used. AT-MSCs are the most common type of MSC studied so far, and they have the capacity to be differentiated into all three layers of the cornea. Most of the MSC differentiation studies have targeted corneal epithelial as the differentiation outcome.

In regard to corneal epithelial cell differentiation, the chemical induction method is more commonly studied compared to co-culture. The most common small molecules that supplement the chemical induction method are BMP4, ATRA, and EGF. In addition, CK3 and CK12 are the most common markers that are always used to evaluate the extent of the corneal differentiation process, as summarized in Table 1.

## 5. Discussion

Adipose tissue has been found to be more favorable as a source of stem cells in corneal differentiation studies. Subcutaneous adipose tissue procurement during liposuction procedures generates an adequate quantity of cells for extensive differentiation studies [85]. Nevertheless, ADSCs can be isolated from both subcutaneous and visceral fat tissue depots. Recent studies have shown that ADSCs from visceral fat express higher stemness genes like *c-MYC*, *SOX2*, *KLF4*, and *NANOG* compared to those isolated from subcutaneous fat. In addition, the expression of the stemness gene in visceral ADSC also translated with a higher number of positive adipocyte and osteocyte differentiated cells following multilineage differentiation [50,86].

The chemical induction method utilizes small molecules to induce corneal differentiation. Different chemical cocktails drive MSCs toward various corneal cell types such as corneal epithelial, stroma, and endothelial cells (Figure 2). BMP4 and ATRA induction have demonstrated effectiveness in promoting corneal epithelial differentiation, while fibroblast growth factor (FGF) is more associated with corneal stroma [52,64]. Additionally, the co-culture method with corneal epithelial cells or limbal stem cells effectively differentiates MSCs toward somatic corneal tissue. The co-culture method aims to replicate the physiological signaling that occurs in vivo. However, this method relies on donated corneal cells from human donors [77,78,79,84].

Current strategies for MSC differentiation are moving toward 3D culture systems to mimic the human tissue environment. Scaffold-based cultures using hydrogels are one of the few types of 3D culture systems applied for MSC differentiation [87]. Bioengineered 3D scaffolds can be constructed from biomaterials such as collagen, hyaluronic acid, fibrin, and agarose to provide an extracellular matrix (ECM) environment that mimics the corneal tissue niche that cannot be achieved in a 2D culture system [77,82]. Chemical induction and co-culture differentiation methods in combination with a 3D co-culture system using biomaterials can be deployed in the future development of MSC corneal differentiation.

Espandar et al., in 2012, used hyaluronic acid (HA)-derived scaffolds to differentiate ADSCs into keratocytes in animal models. In 2014, Garzon et al. incorporated a fibrin–agarose scaffold to support WJ-MSC differentiation into corneal epithelial cells. Furthermore, Soleimanifar et al. in 2017 utilized a hybrid of polyurethane (PU) and silk materials to make a nanofibrous scaffold as a 3D culture system to differentiate CJ-MSCs toward corneal epithelial cells. The authors of those studies used different types of biomaterials to construct a 3D culture system for corneal differentiation in different types of MSCs, as presented in Table 1 [74,77,83].

## 6. Conclusions

To date, MSCs have been used in differentiation toward corneal epithelial, stromal, and endothelial cells. ADSCs are the most common type of MSC that has been used for corneal differentiation. Corneal epithelial cells are the most common corneal cell type targeted by researchers for corneal differentiation. Chemical induction with small molecules, especially BMP4, ATRA, and EGF, has gained more popularity in corneal epithelial cell differentiation. An increasing number of studies have utilized 3D culture systems using hydrogels to accelerate corneal differentiation as it provides an extracellular environment for MSCs. Moving forward, research should focus on exploring the synergistic effects of combining small molecules with advanced 3D culture systems in the corneal cell differentiation of MSCs. Long-term studies are also needed to assess the efficacy of corneal epithelial tissue derived from MSCs in animal models.

## Figures and Tables

**Table 1:** Summary of MSC differentiation studies on corneal tissue.

No	Corneal Cell Differentiation	MSC Source	Method of Differentiation	Differentiation Markers	Outcomes
1	Cornea epithelial [53]	AT-MSCs	Induced by conditioned medium (derived from human corneal epithelial cell and limbal fibroblast culture)	CK3 and CK12	Increase in the gene and protein expression of CK3 and CK12
2	Cornea epithelial [79]	AT-MSCs	Co-cultured with limbal epithelial stem cells for 14 days	*CK3*, *CK12*, *p63*, and *ABCG2*	Significant increase in *CK3* expression
3	Cornea epithelial [66]	AT-MSCs	Induced for 12 days with mesenchymal–epithelial transition (MET) medium/MesenPRO-RS™ (50 μM VPA, 300 nM CHIR99021, 1 μM RepSox, 5 μM Tranylcypromine, 500 nM A-83-01, 10 μM ATRA, 2% serum, and 1% AA)	CK3, CK12, and CDH1	Increase in the gene and protein expression of CK3 and CK12
6	Cornea epithelial [67]	BM-MSCs	Induced for 4 days by induction media (ATRA, BMP-4, and EGF) and 9 days by differentiation media (500 ng/mL hydrocortisone, 5 μg/mL insulin, and 10 ng/mL hEGF)	p63, CK8 (ectodermal lineage), and CK3, CK12 (corneal epithelial cell markers)	Increase in the gene expression of *CK3*, *CK8*, and *CK12*. Increase in the protein expression of CK3, CK8, and CK12
7	Cornea epithelial [73]	BM-MSCs	Induced for 12–14 days by induction media (5 g/mL insulin, 0.18 mM adenine, 0.4 g/L hydrocortisone, 10^–10^ M cholera toxin, 2 × 10^–9^ M triiodothyronine, and 10 ng/mL EGF)	B1-integrin, CEBPd, ABCG2, p63, and CK3	Increase in the gene expression of *B1-integrin*, *CEBPd*, *ABCG2*. Increase in the protein expression of p63 and CK3
8	Cornea epithelial [68]	BM-MSCs	Induced for 7 days by differentiation media (DMEM, 2% FCS, 1% antibiotic, and 1 μM of all-trans-retinoic acid)	CK3, CK12, CK19, E-cad, and ITGB1	Increase in the gene expression of *CK3*, *CK12*, *CK19*, *E-cad*, and *ITGB1* compared to the control. Increase in the protein expression of CK12 and CK19 compared to the control
9	Cornea epithelial [74]	CJ-MSCs	Cultured in nanofibrous scaffolds for 12 days with differentiation medium (DMEM:F12, 5% FCS, 1% Pen-Strep, 500 ng/mL hydrocortisone, 5 µg/mL insulin, 2 nM triiodothyronine, adenine, and 10 ng/mL recombinant human EGF)	CK 3, 8, 12, DSG1, and DSC1	Increase in the expression of all epithelial genes in the co-culture system compared to the control Higher CK3 protein expression in the treated group compared to the negative control
10	Cornea epithelial [75]	CJ-MSCs	Co-cultured with corneal epithelial cells for 12 days in differentiation medium (DMEM:F12, 5 ng/mL of EGF, 5 μg/mL of insulin, 5 μg/mL of transferrin, 5 ng/mL of sodium selenite, 0.5 μg/mL of hydrocortisone, 30 ng/mL of cholera toxin A, 0.5% of DMSO, 50 μg/mL of gentamicin, 1.25 μg/mL of amphotericin B and 5% of FBS)	*Involucrin*, *nestin*, *ABCG2*, *Np63 alpha*, *DSG1*, *DSC1*, and *CK 3*, *8*, *12*, *14*, and *15*	Increase in the expression of all epithelial genes in the co-culture system compared to negative control
11	Cornea epithelial [78]	Human Exfoliated Deciduous Teeth (HED)-MSCs	Co-cultured for 21 days with a human corneal epithelial cell line	CK3 and CK19	Increase in the gene expression of *CK3* and *CK19*. Increase in the protein expression of CK3 and CK19
12	Cornea epithelial [77]	WJ-MSCs	Co-cultured with keratocyte in a hydrogel scaffold 3D system	CK3, CK12, PKG, ZO1, and CX43	Increase in the protein expression of CK3, CK12 and CX43
13	Corneal stroma [72]	AT-MSCs	Chemical induction (DMEM, 10 ng/mL fibroblast growth factor 2 (FGF2), 0.1 mM ascorbic acid-2-phosphate (A2P), and heparin-stripped, platelet-poor horse serum (HSHS))	Keratocan, keratan sulfate, and aldehyde dehydrogenase 3 family (ALDH3A1)	Increase in the gene and protein expression of keratocan, keratocan sulfate, and ALDH3A1
14	Corneal stroma [83]	AT-MSCs	Cultured in hydrogels and supplemented with 5 ng/mL bFGF for 14 days	ALDH and keratocan	Increase in the phenotype of corneal stroma
15	Corneal stroma [84]	AT-MSCs	Co-cultured for 16 days with keratocytes in keratocyte differentiation media (KDM)	Keratocan, ALDH3A1, cadherin 5 (CDH5), and alpha-smooth muscle actin (αSMA)	Increase in the protein expression of ALDH3A1 and keratocan
16	Cornea endothelial [71]	AT-MSCs	Induced for 10 days (DMEM:F12, 500 ng/mL Noggin, 10 uM SB431542, 20% KSR, 1 mM L-glutamine, and 8 ng/mL of FGF2) Differentiated in 20 days (basal media, 0.1X B27, 10 ng/mL PDGF-BB, and 10 ng/mL DKK-2)	Na^+^/K^+^ ATPase, ZO1, and Aquaporin1	Increase in the gene and protein expression of Na^+^/K^+^ ATPase, ZO1, and Aquaporin1

## Data Availability

No new data were created or analyzed in this study. Data sharing is not applicable to this article.
